# Multi-Band Up-Converted Lasing Behavior in NaYF_4_:Yb/Er Nanocrystals

**DOI:** 10.3390/nano8070497

**Published:** 2018-07-05

**Authors:** Ya-Pei Peng, Wei Lu, Pengpeng Ren, Yiquan Ni, Yunfeng Wang, Peiguang Yan, Yu-Jia Zeng, Wenfei Zhang, Shuangchen Ruan

**Affiliations:** 1 Shenzhen Key Laboratory of Laser Engineering, Key Laboratory of Advanced Optical Precision Manufacturing Technology of Guangdong Higher Education Institutes, College of Optoelectronic Engineering,, Shenzhen University, Shenzhen 518060, China; yypeng@szu.edu.cn (Y.-P.P.); ncurenpeng@outlook.com (P.R.); 18394667281@163.com (Y.N.); yanpg@szu.edu.cn (P.Y.); yjzeng@szu.edu.cn (Y.-J.Z.); 2 University Research Facility in Materials Characterization and Device Fabrication, The Hong Kong Polytechnic University, Hong Kong 999077, China; wei.lu@polyu.edu.hk; 3 Department of Applied Physics, The Hong Kong Polytechnic University, Hong Kong 999077, China; heybabyruthless@hotmail.com

**Keywords:** random laser, nanomaterials, up-conversion

## Abstract

Random lasers have attracted great interests and extensively investigation owing to their promising applications. Here, we explored unambiguously the multi-band up-converted random lasing from NaYF_4_:Yb,Er nanocrystals (NCs). NaYF_4_:Yb,Er NCs exhibit high effective up-conversion luminescence when they are excited by continuous wave 980 nm laser. We investigated a planar microcavities approach wherein the NaYF_4_:Yb,Er NCs showed up-converted lasing behavior. The optical pumping of NaYF_4_:Yb,Er NCs by 980 nm pulsed laser excitation exhibited multi-band lasing. The NaYF_4_:Yb,Er NCs showed multi-band lasing emission with a line width of 0.2 nm at 540 nm and 0.4 nm at 660 nm. This research promotes potential application in bioimaging and biomedical fields.

## 1. Introduction

**Figure 1:**
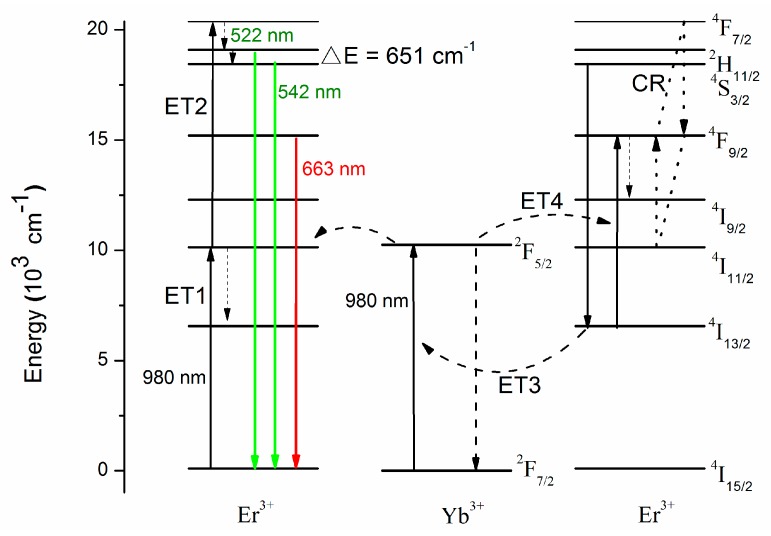
Schematic diagram of energy levels and transitions of Yb^3+^ and Er^3+^ ions by 980 nm pumping.

Up-conversion luminescence of rare-earth ions doped fluoride based nanocrystals (NCs) has garnered significant interest recently due to their multiple potential applications including laser source, color display, biomedical imaging, three-dimensional display, drug-carrier systems, optical devices, and solar cells [1,2,3,4,5,6,7,8,9,10]. Up-conversion luminescence is the process that luminescent NCs convert near-infrared (NIR) light to visible light by emitting high-frequency photons after absorbing low-frequency photons [11]. The absorption and scattering of NIR radiation is quite low in biological tissues, concomitantly with low levels of background autofluorescence, as optical transparency in NIR region of biological tissues can be large, which is within the “optical transparency window” of tissue. Therefore, it possesses high light penetration depth in tissues, high sensitivity, less photo bleaching, weak autofluorescence, and no photo damage to biological specimens, which are useful for bioimaging applications [11]. Besides, the up-conversion luminescence which absorbs two or more photons is a nonlinear optical process, and so it is easy to be quantum-coherently controlled [2,12,13]. High light intensity is preferred in bio-applications to monitor the cellular entry pattern of a drug and destroy certain viruses or cancer cells in vivo [14,15,16]. Therefore, up-converted lasing is one of the most feasible ways to enhance luminescence intensity and simultaneously maintain low levels of background. Among all kinds of up-conversion nanomaterials, hexagonal-phase NaYF_4_:Yb,Er NCs have been demonstrated to be one of the most efficient NIR-to-visible up-converting materials, which have a lower photon energy (≈350 cm^−1^) and a higher refractive index [16,17,18]. Furthermore, the investigation of NaYF_4_ can be quite economical due to their facile synthesis methods and accessible raw materials. The up-conversion mechanism of NaYF_4_:Yb,Er NCs is depicted in Figure 1. The up-conversion hosts can yield emissions in green (520 and 540 nm) and red (650 nm) colors by a continuous wave (CW) 980 nm diode laser pumping, corresponding to transitions from energy levels ^2^H_11/2_, ^4^S_3/2_, and ^4^F_9/2_ to the ground state ^4^I_15/2_ of Er^3+^ ions, respectively. The up-conversion luminescence included in multi-step energy transfer (ET) of excited state Er^3+^ ions, excited state absorption (ESA), and the continuous energy-transfer up-conversion between Er^3+^ ions and Yb^3+^ ions. Furthermore, the luminescence integrated intensity ratio of the red to green regions strongly depends on the presence of Yb^3+^ ions because the energy-transfer up-conversion (ETU) process is dominant in the samples. The presence of Yb^3+^ ions on NCs leads to more efficient absorption of exciting light at 980 nm and increases the efficiency of energy transfer. At first step of the excitation, the excitation at 980 nm of the Yb^3+^ sensitizer provokes a pump photon from the ^2^F_7/2_ ground state to the ^2^F_5/2_ excited state. Then the excited Yb^3+^ ion transfers its energy to a neighbor Er^3+^ ion and depopulates the ground state, simultaneously. The transferred energy promotes Er^3+^ ion transition from the ^4^I_15/2_ ground state to the ^4^I_11/2_ excited state. Moreover, the Er^3+^: ^4^I_11/2_ level can receive another laser photon, and then transits to the higher energy level ^4^F_7/2_, or non-radiatively relaxes to the ^4^I_13/2_ level of Er^3+^ ions. According to a Boltzmann distribution, the photons at the ^4^F_7/2_ level relax directly to the ^2^H_11/2_ and ^4^S_3/2_ levels and then transfer back to the ground state of ^4^I_15/2_, hence bringing about 522 nm and 542 nm emissions.

Recently, random lasers have attracted great interests for researchers because they can be amplified by multiple scatterings in a disorder system [19]. Random lasing is desired in nanostructures through surface nanoparticle amplification. The random laser is a microcavity whose feedback came from confusion-induced scattering. When gain exceeds loss, along with population inversion and simulated emission, random lasing can be obtained. Random laser has some excellent feasible applications, such as biological probe, display, and speckle-free images, due to its advantages including simple design, easy fabrication, compactness, low threshold, angle-free emission, and low cost, compared to complicated structure such as photonic band gap microcavities [20,21,22,23]. Therefore, we designed the planar microcavity to demonstrate the random laser action. Integrated device and simple technology is useful for further applications.

In this study, we synthesized uniform NaYF_4_:20%Yb,2%Er NCs via a solvothermal method [17,24] and systematically investigated their up-conversion emission properties at room temperature. Effective up-conversion luminescence of NaYF_4_:20%Yb,2%Er NCs excited by CW 980 nm laser was obtained. Moreover, the NaYF_4_:20%Yb,2%Er NCs film was used as a laser gain medium to demonstrate random laser by planar microcavities, which maintain stronger optical confinement of optical modes with lower cavity losses [25]. The nanosecond pulsed laser with high peak intensity is used to achieve high optical gain from the NaYF_4_:20%Yb,2%Er NCs. Enhancement and suppression of spontaneous emission in micro cavities are demonstrated in variety of optical materials such as organic dye films and solutions, semiconductors, and quantum dots [25,26,27]. Therefore, this work facilitates the use of NaYF_4_:20%Yb,2%Er NCs in bio-imaging materials.

## 2. Materials and Methods

High-quality NaYF_4_:20%Yb,2%Er NCs were synthesized through a typical solvothermal method [17]. All chemical reagents were analytical grade and used without further purification. For a typical synthesis process: YCl_3_ (0.1523 g), YbCl_3_ (0.0558 g), and ErCl_3_ (0.0054 g) were mixed with 3 mL oleic acid (OA) and 15 mL 1-octadecene (ODE) in a 100 mL flask and heated to 160 °C for 30 min to form a homogeneous solution, and then cooled down to 50 °C. After that, 10 mL methanol solution containing NaOH (0.1 g) and NH_4_F (0.15 g) were added into the flask and stirred quickly for 30 min in 50 °C. Subsequently, the solution was heated to 120 °C for 30 min to completely evaporate methanol, and then heated to 300 °C for 1 h protected by argon atmosphere. After the solution was naturally cooled down to room temperature, nanocrystals were precipitated from the solution with ethanol. Then, the precipitates were washed three times with ethanol and water (1:1 *v*/*v*) mixture. Finally, the NCs were dispersed in cyclohexane for optical measurements.

The morphology of the NaYF_4_:Yb/Er NCs was characterized by a JEOL JEM-2100F high-resolution transmission electron microscope (HR-TEM, Tokyo, Japan). X-Ray powder diffraction spectra of NCs were measured by a Rigaku SmartLab Intelligent X-ray diffractometer (XRD, Austin, TX, USA ) with filtered Cu Kα radiation (λ = 1.5406 Å, operating at 45 kV and 200 mA). Fluorescence spectra were measured by a HORIBA iHR320 fluorescence spectrophotometer (Minami-ku, Kyoto, Japan) under CW 980 nm laser pumping. Lasing characteristics of NCs were studied by third harmonic generation from a neodymium-doped yttrium aluminum garnet (Nd:YAG) pulsed laser (355 nm wavelength, 6 ns pulse width, 10 Hz frequency, Continuum Surelite, San Jose, CA, USA) with an optical parameter oscillator (Continuum Horizon, San Jose, CA, USA) to expand the Nd:YAG laser to the excitation wavelengths at 980 nm. The lasing emission spectra are not modified by data processing software. The laser beam was focused onto the sample by an optical lens with a focal length of 50 mm and laser spot diameter was 800 μm. All of the measurements were conducted at room temperature.

## 3. Results

### 3.1. Morphology and Structural Characterization

**Figure 2:**
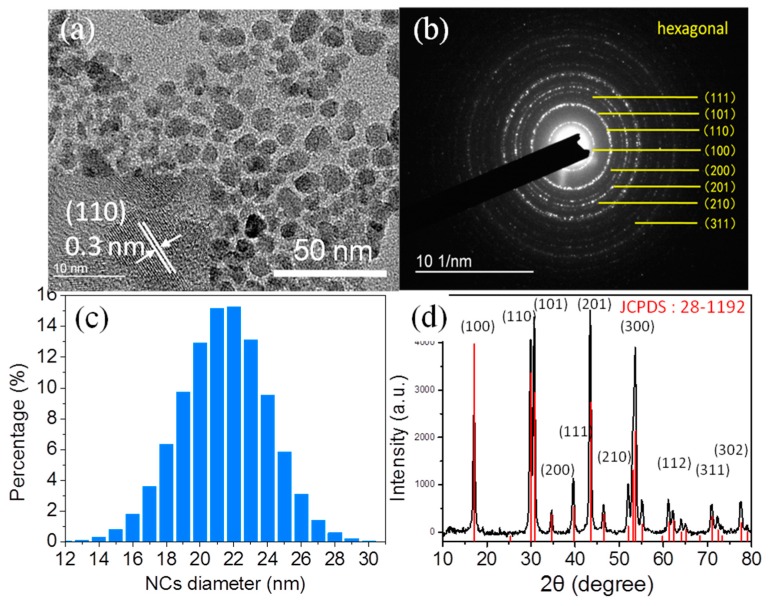
(**a**) TEM image and HR-TEM image (inset); (**b**) SAED pattern; (**c**) size distribution; and (**d**) XRD pattern of the NaYF_4_:Yb,Er NCs.

The transmission electron microscopy (TEM) images and HR-TEM images of the NaYF_4_:Yb,Er NCs are shown in Figure 2a. It is observed that the NaYF_4_:Yb,Er NCs are nearly spherical in shape and uniformly distributed. From the HR-TEM image, we can clearly distinguish lattice fringes on the individual crystals indicating that the NCs are highly crystalline. The lattice spacing of the NCs was measured to be about 0.3 nm, which corresponds to a (110) lattice facet of the hexagonal NaYF_4_ structure. This result is consistent with the results of the selected area electron diffraction (SAED) pattern [28,29]. The SAED and size distribution of NCs are given in Figure 2b,c, respectively. The SAED pattern of the NCs can be indexed to the (100), (110), (101), (200), (111), (201), (210), (002), (300), (211), (112), (220), (202), (310), (311) and (320) planes of the standard hexagonal β-NaYF_4_ structure (JCPDS: 28-1192) [30,31]. The NaYF_4_:Yb,Er NCs with size distribution between 16 and 26 nm and average size about 22 nm without aggregation is observed and analyses from TEM images by Gatan DigitalMicrograph software (GMS 3, Pleasanton, CA, USA). In addition to morphology and grain size, the crystalline phase of NaYF_4_:Yb,Er NCs is also a crucial issue. The XRD patterns of NaYF_4_:Yb,Er NCs (black line patterns) are shown in Figure 2d, which evidently demonstrate that the sample was highly crystalline in nature. The red line pattern is given according to the standard power diffraction file (PDF) 28-1192, provided by the Joint Committee on Powder Diffraction Standards (JCPDS). The peak positions and intensities of these sample pattern match well and closely correspond to the reported and calculated patterns for hexagonal β-NaYF_4_ [30,32,33,34]. The corresponding (*h k l*) values are given above. No cubic phase diffraction peaks or other impurities were observed. The observed broad diffraction peaks are an indication of the small size of the NCs. According to the line broadening of the diffraction peak of the NaYF_4_:Yb,Er NCs, an average crystallite size of 25 nm was calculated by using the Debye–Scherrer formula, which closely matches to the particle size determined from the TEM software analyses.

### 3.2. Up-Conversion Luminescence Properties

**Figure 3:**
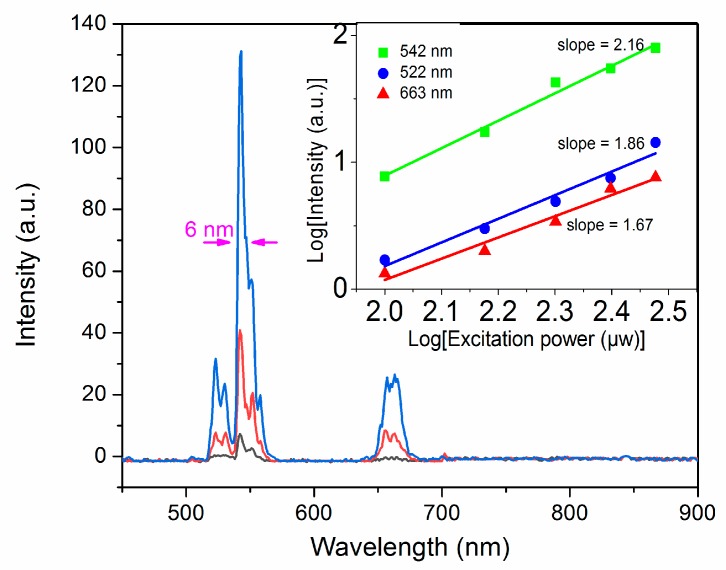
Up-conversion luminescence spectra of NaYF_4_:20%Yb,2%Er by 980 nm excitation at room temperature. The inset is the log-log plots of emission intensity of different emission bands versus excitation power for the NCs.

The up-conversion luminescence spectra of NaYF_4_:Yb,Er NCs with different pump power under CW 980 nm excitation at room temperature is shown in Figure 3. According to the energy level diagram in Figure 1, there were three distinct emission peaks centered at 522, 542, and 663 nm, which correspond to the transitions between energy levels ^2^H_11/2_, ^4^S_3/2_, and ^4^F_9/2_ to ^4^I_15/2_ of Er^3+^ ions, respectively. It is observed that the up-conversion emission intensity increases with the increase of the excitation power at 980 nm. The up-conversion emission intensity ($I_{up}$) can generally be expressed as [16,35,36]:

(1) $$I_{up}\infty P_{ex}^{N},$$

where $I_{up}$ is the up-conversion luminescence intensity, $P_{ex}^{}$ is the excitation power, and *N* is the absorbed photon numbers for producing one up-conversion emission photon. It can be obtained from the slope of the fitted line of the plot of log($I_{up}$) versus log($P_{ex}^{N}$) at low excitation density. It should be noted that the “*N*” values can be affected by the competition process between the up-conversion rate and the decay rate at the intermediate states at high excitation density [37]. As shown in the inset of Figure 3, the slopes of the linear fits, *N* values, are 2.16, 1.86, and 1.67 for the up-conversion emissions at 542, 522, and 663 nm in the NaYF_4_:20%Yb,2%Er NCs, respectively. The green up-conversion emission is realized through the excited first photon to the ^4^I_11/2_ of Er^3+^ ion via energy transfer from neighboring Yb^3+^ ion. Immediately following this process, the excited photon at ^4^I_11/2_ is further excited to ^4^F_7/2_ state by another energy transfer from Yb^3+^ ion or excited state absorption by the second photon excitation. According to the Miyakawa–Dexter theory, the probability of phonon-assisted energy transfer can be expressed by [38]:

(2) $$Wij=W(0)e^{-\alpha \Delta E},$$

where W(0) and α are constants determined by the host and Δ*E* is the energy gap between the transitions involved in the phonon-assisted energy transfer. The energy gap between ^2^H_11/2_ and ^4^S_3/2_ is quite small, resulting in the nonradiative transition. Therefore, the slope of the fitted line at 542 nm is higher than the slope at 522 nm from the inset of Figure 3. These results indicate that the Er^3+^: ^2^H_11/2_→^4^I_15/2_ (522 nm), Er^3+^: ^4^S_3/2_→^4^I_15/2_ (542 nm), and Er^3+^: ^4^F_9/2_→^4^I_15/2_ (663 nm) up-conversion emissions process are two-photon absorption processes on the NaYF_4_:20%Yb,2%Er NCs.

### 3.3. NaYF_4_:Yb,Er NCs Up-Converted Random Lasers

**Figure 4:**
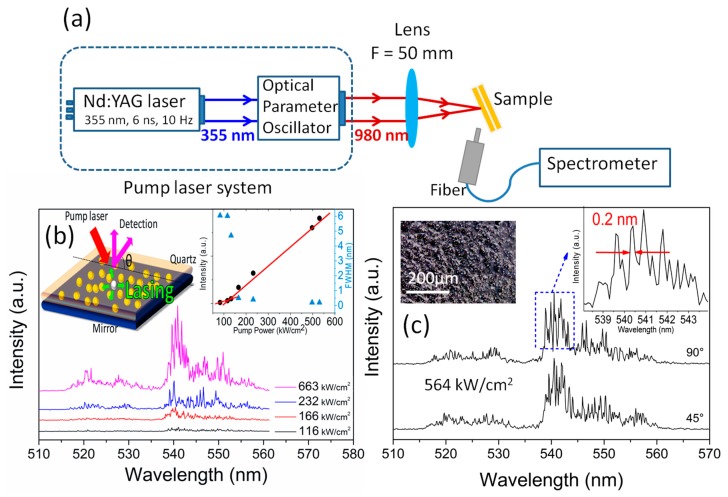
(**a**) Experimental setup of random laser system of NCs; (**b**) Emission spectra versus different excitation power. The left inset is the schematic of the proposed NaYF_4_:Yb,Er NCs lasers. The right insets are the corresponding input–output curve and FWHM; (**c**) Emission spectra of NaYF_4_:20%Yb,2%Er NCs film at around 540 nm wavelength recorded under 980 nm nanosecond laser excitation at different observation angles, θ. The left inset is the optical microscope image of the NaYF_4_:20%Yb,2%Er NCs film. The right inset is the FWHM of the emission spectra of NCs laser.

The synthesized NaYF_4_:20%Yb,2%Er NCs can be used as optical gain medium to realize random lasers. The experimental setup of a random laser of NaYF_4_:20%Yb,2%Er NCs is shown in Figure 4a. We designed planar microcavities which sandwich the NaYF_4_:Yb,Er NCs film between two reflectors. The left inset of Figure 4b shows the schematic of the proposed NaYF_4_:Yb,Er NCs lasers. The NaYF_4_:Yb,Er NCs is solidified to form a film of about 300 μm thickness and sandwiched between a quartz plate and an Aluminum (Al) mirror (Al coated glass substrate). The mirrors are used to improve the longitudinal confinement of light and achieve optical feedback along the laser microcavity. The laser characteristics of the NaYF_4_:Yb,Er NCs film can be examined by using a 980 nm nanosecond laser excitation. The laser beam is focused onto a spot of 800 μm in diameter on the NCs film through the quartz mirror. The small beam size promotes the lateral confinement of the emission light from the NCs film so that a planer microcavity can be formed [25]. Laser emission is detected from the side of quartz mirror. A plot of emission spectra of NCs laser at room temperature versus different excitation power is shown in the Figure 4b. The input–output curve and full width at half maximum (FWHM) are shown in the right inset of Figure 4b. A broad spontaneous emission band centered at ≈540 nm is observed for the NCs film when the excitation power is below an excitation threshold value of ≈125 kW/cm^2^, namely kink of the input–output curve. The excitation threshold in our works is lower than those of random lasing without planar microcavity [39,40,41]. The FWHM is acutely decreasing from 6 to 0.2 nm with increasing of pump power. In addition, more sharp peaks further emerge from the emission spectra with increasing pump power. Due to the coherent optical feedback provided by the NaYF_4_:Yb,Er NCs to form the closed light loop path, the sharp peaks represent the realization of lasing. It also can be observed in Figure 4b that the lasing modes are randomly distributed in the lasing spectra. This is because the NaYF_4_:Yb,Er NCs are aggregated with each other in the gain film after solvent evaporation. The aggregation leads to light scattering in the gain medium [42]. This phenomenon can also be verified by different lasing spectra obtained from different detection angles because the NaYF_4_:Yb,Er NCs are distributed randomly inside the film (Figure 4c). As shown in Figure 4c, the lasing spectra do not reveal the presence of Fabry–Perot modes as the mode spacing is distributed non-uniformly over the emission spectrum. The left inset of Figure 4c shows the optical microscope image of the NCs film. It is observed that there are plenty of NaYF_4_:Yb,Er NCs, which can satisfy the sufficient scattering between NCs and NCs to realize random lasing. Moreover, sharp peaks with FWHM less than 0.2 nm emerge from the emission spectrum when the excitation power larger than that of the threshold value, as shown in right inset of Figure 4c. The FWHM of lasing peak is less than that in other reports [43,44]. The Q factor of the NaYF_4_:Yb,Er microcavity can be approximately defined as Q = λ_p_/Δλ [45]. λ_p_ and Δλ are sharp peak wavelength and FWHM, respectively. The Q factor of NaYF_4_:Yb,Er microcavity is about 2700, which is comparable with other random laser systems [43,46,47]. As a result, it is verified that NaYF_4_:Yb,Er NCs film supports ultrahigh Q coherent random laser microcavity with low threshold.

**Figure 5:**
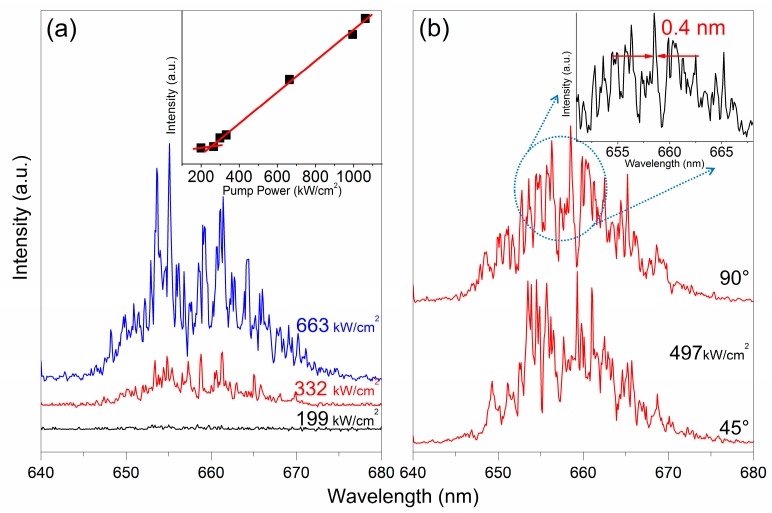
(**a**) Emission spectra of NaYF_4_:20%Yb,2%Er NCs film at around 660 nm wavelength versus different excitation power under 980 nm nanosecond laser excitation. The inset shows the corresponding input–output curve; (**b**) Emission spectra versus observation angle, θ. The inset shows the FWHM of the NaYF_4_:20%Yb,2%Er NCs laser.

The plots of the lasing spectra versus different excitation power at around 660 nm of the planer microcavity by using the 980 nm nanosecond laser pumped is shown in Figure 5a. The corresponding input–output curve is shown in the inset of Figure 5a. The emission spectra versus observation angle, θ, of the NaYF_4_:20%Yb,2%Er NCs film around 660 nm is shown in Figure 5b. As the pump power exceeds an excitation threshold value of ≈254 kW/cm^2^, sharp peaks emerge from the emission spectra with a line width of 0.4 nm, as shown in the inset of Figure 5b. The excitation threshold value at 660 nm emission is larger than that of at 540 nm emission due to the lower fluorescence efficiency at 660 nm.

## 4. Conclusions

We have demonstrated multi-band up-conversion random lasing from NaYF_4_:Yb,Er NCs. It is noted that lasing emission with a peak wavelength of ≈540 nm and 660 nm under 980 nm nanosecond excitation is obtained from the NaYF_4_:20%Yb,2%Er NCs film sandwiched between an Al mirror and a quartz mirror. This is because longitudinal optical confinement is achieved via the optical feedback between the two interface, and lateral optical confinement of high-Q random microcavities is achieved through the non-uniform distribution of NCs. Hence, the formation of a low loss planar microcavity can support the random lasing action at room temperature. Discrete sharp peaks, representing the formation of a closed light loop path, with FWHM of 0.2 nm at 540 nm and 0.4 nm at 660 nm, are achieved from the emission spectra. The variation of the emission spectra with different detection angles verified the support of random lasing action. As a result, our proposed NaYF_4_:20%Yb,2%Er NCs, which have been verified unambiguously the realization of up-conversion random lasing, are potential optical gain mediums suitable for the optical and biological applications.
